# Respiratory syncytial virus–related lower respiratory tract infection hospitalizations in infants receiving nirsevimab in Galicia (Spain): the NIRSE-GAL study

**DOI:** 10.1007/s00431-025-06151-3

**Published:** 2025-05-02

**Authors:** Angela Manzanares, Jacobo Pardo-Seco, Irene Rivero-Calle, Ana Dacosta-Urbieta, Narmeen Mallah, María-Isolina Santiago-Pérez, Olaia Pérez-Martínez, María-Teresa Otero-Barrós, Nuria Suárez-Gaiche, Rolf Kramer, Jing Jin, Leticia Platero-Alonso, Rosa-María Alvárez-Gil, Olga-María Ces-Ozores, Victoria Nartallo-Penas, Susana Mirás-Carballal, Marta Piñeiro-Sotelo, Juan-Manuel González-Pérez, Carmen Rodríguez-Tenreiro-Sánchez, Antonio Salas, Carmen Durán-Parrondo, Federico Martinón-Torres

**Affiliations:** 1 Translational Pediatrics and Infectious Diseases, Hospital Clínico Universitario de Santiago (SERGAS) and University of Santiago de Compostela (USC), Santiago de Compostela, Galicia, Spain; 2 Genetics, Vaccines and Infectious Diseases Research Group (GENVIP), Instituto de Investigación Sanitaria de Santiago de Compostela (IDIS), Santiago de Compostela, Galicia, Spain; 3 WHO Collaborating Centre for Vaccine Safety, Santiago de Compostela, Galicia, Spain; 4 Centro de Investigación Biomédica en Red de Enfermedades Respiratorias (CIBERES), Instituto de Salud Carlos III, Madrid, Spain; 5 Genética de Poblaciones en Biomedicina (GenPoB) Research Group, Instituto de Investigación Sanitaria (IDIS), Hospital Clínico Universitario de Santiago (SERGAS), Galicia, Spain; 6 Department of Preventive Medicine, University of Santiago de Compostela (USC), Santiago de Compostela, Galicia, Spain; 7 Instituto de Psicología (IPsiUS), University of Santiago de Compostela (USC), Santiago de Compostela, Spain; 8 Department of Epidemiology, Dirección Xeral de Saúde Pública, Consellería de Sanidade, Xunta de Galicia, Galicia, Spain; 9 Sanofi Vaccines, Lyon, France; 10 Department of Communicable Diseases, Dirección Xeral de Saúde Pública, Consellería de Sanidade, Xunta de Galicia, Galicia, Spain; 11 Galician Center for Disease Control and Prevention, Dirección Xeral de Saúde Pública, Consellería de Sanidade, Xunta de Galicia, Galicia, Spain; 12 Subdirección de Sistemas y Tecnologías de La Información, Xunta de Galicia, Consellería de Sanidade, Galicia, Spain; 13 Unidade de Xenética, Instituto de Ciencias Forenses, Facultade de Medicina, Universidade de Santiago de Compostela (USC), Galicia, Spain; 14 Dirección Xeral de Saúde Pública, Consellería de Sanidade, Xunta de Galicia, Galicia, Spain

**Keywords:** Respiratory syncytial virus (RSV), Low respiratory tract infection (LRTI), Nirsevimab, Breakthrough cases, Immunization, Infants

## Abstract

As part of the NIRSEGAL study (https://www.nirsegal.es/en), we present the clinical characteristics and course of respiratory syncytial virus (RSV)-related low respiratory tract infection (LRTI) hospitalizations in infants eligible for nirsevimab administration during the 2023–2024 season. Infants eligible for nirsevimab immunization (born between 1 April, 2023, and 30 March, 2024) who were hospitalized due to RSV-related LRTI between September 25, 2023, and April 15, 2024, in a hospital from the Galician Public Health system were included. Clinical and demographic characteristics of RSV-related LRTI hospitalizations were analyzed, with comparisons made between breakthrough cases (those immunized with nirsevimab) and non-breakthrough cases. During the study period, 69 RSV-related LRTI hospitalizations were recorded, with a median hospital stay of 4 (interquartile range (IQR) 3–6) days; 65.2% (*N* = 45) were breakthrough cases. The median age was 2.7 (IQR 1.5–5.2) months, and more than half of them (*N* = 39, 56.5%) were male. The incidence of cases was parallel to the RSV epidemic curve, suggesting no waning of nirsevimab efficacy. Of the total hospitalizations, 16 infants (23.2%) had a high-risk condition, 44 (63.8%) needed oxygen support, 15 (21.7%) were admitted to the intensive care unit (ICU), and 11 (15.9%) received non-invasive mechanical ventilation (NIMV). No statistically significant differences were observed in these characteristics when comparing breakthrough and non-breakthrough cases. *Conclusion*: In the nirsevimab era, a substantial proportion of children who were hospitalized for RSV-related LRTI needed oxygen support, NIMV, and ICU admission. Clinical characteristics, timing, and outcomes were comparable between breakthrough and non-breakthrough cases.

*Trial registration*: The NIRSE-GAL study protocol was registered on ClinicalTrials.gov (NCT06180993).

**Table:** 

**What is Known**: • *Nirsevimab, a long-acting monoclonal antibody, has shown high effectiveness in preventing RSV-related hospitalizations and has been included in some European countries’ immunization programs*.	
**What is New:** • *During the first RSV season after the universal implementation of nirsevimab in Galicia (Spain), a large proportion of hospitalized infants had high-risk conditions, yet disease severity markers (oxygen need, ICU admission, NIVM) were comparable between breakthrough and non-breakthrough cases*. • *No signal of waning protection over time was observed among breakthrough cases, reinforcing the potential value of early, season-wide immunization*.

**Supplementary Information:**

The online version contains supplementary material available at 10.1007/s00431-025-06151-3.

## Introduction

Nirsevimab (Beyfortus, AstraZeneca [Södertälje, Sweden], and Sanofi [Gentilly, France]) was approved by the European Medicines Agency in October 2022. Galicia, a region with approximately 14,500 annual births [1] and 540 annual respiratory syncytial virus (RSV)-related low-respiratory-tract infection (LRTI) hospitalizations in infants [2], was among the first to introduce nirsevimab as part of the routine immunization program [3]. The NIRSE-GAL study [4] demonstrated this measure to reduce RSV-related LRTI hospitalizations by 89% in Galicia in the 2023–2024 season [5], as later did other studies in Europe [6–11]. This raises the need to update knowledge on current RSV-related LRTI-related hospitalizations.

Here, as part of NIRSE-GAL, we aim to describe the clinical features and course of RSV-related LRTI hospitalizations in infants in the nirsevimab era and to explore the characteristics of the breakthrough cases.

## Methods

### Study design

NIRSE-GAL study is a prospective observational study in the 14 public hospitals included in the Galicia Public Health system (SERGAS), accounting for all pediatric and maternity services (9818 hospital beds) [9]. The detailed protocol has already been published [2].

Firstly, infants eligible for nirsevimab administration (born between Sept 25, 2023, and March 31, 2024, seasonal group, and between April 1 and Sept 24, 2023, catch-up group) who had a hospital admission between September 25, 2023, and April 15, 2024, were automatically identified from the registries “hospital admissions” From these, patients with a positive RSV result identified by microbiology laboratory test during the hospitalization, or within 10 days before admission, were reviewed by public health specialists who decided whether the hospitalizations were RSV-related LRTI (appendix 1). In case of any uncertainty, a second assessment was carried out by an expert advisory committee (EAC). If a consensus was not achieved, the EAC evaluation prevailed. All infants who were hospitalized for at least one night with an RSV-related LRTI were included. Only the first hospitalization episode of each eligible patient during the study period was considered. Infants with nosocomial RSV infections (whose respiratory symptoms started after 48 h of hospitalization) were excluded.

Breakthrough cases had to meet the following criteria: (i) received nirsevimab at least 7 days before admission, (ii) no clinical symptoms compatible with RSV infection as described by the US Centres for Disease Control and Prevention, or an RSV-positive result in the 7 days after nirsevimab administration, and (iii) no RSV-positive test result in the 15 days before immunization. Recipients who did not meet all these criteria were classified as non-breakthrough cases [2].

Data on the following sociodemographic characteristics were collected: sex, age, weight at birth, gestational age, and high-risk condition (prematurity- < 37 weeks of gestational age-, hemodynamically significant congenital heart disease, severe immunosuppression-hematological disease, cancer, immunosuppressive treatment, primary immunodeficiency-, congenital metabolic disease, Down’s syndrome, cystic fibrosis, neuromuscular disease, bronchopulmonary dysplasia, pulmonary disease). Clinical and hospitalization data were also collected: hospital admission and discharge, intensive care unit (ICU) admission, oxygen support, non-invasive mechanical ventilation (NIMV), invasive mechanical ventilation (IMV), and microbiological co-infections. Nirsevimab administration date was retrieved from the patient “vaccination card.” Data extraction and curation were performed by Galician Health Information Systems experts following predefined procedures.

### Statistical analysis

Demographic and clinical characteristics were descriptively analyzed for the overall sample and stratified by breakthrough status. For continuous variables, median, interquartile range (IQR)were calculated. Statistical significance for continuous variables was explored using the Wilcoxon test. Categorical variables were represented by the number of patients and percentages, and statistical significances were analyzed using the exact Fisher test. There are no missing data for any observation, except for the variable “length of stay in ICU,” which is missing for children who were not admitted to the ICU. In this case, descriptive values and statistical tests were obtained from the observed data.

### Ethical considerations

The NIRSE-GAL study protocol was approved by the ethics committee of Galicia (CEIC 2023–377) and developed in compliance with the International Council for Harmonization Guidelines for Good Clinical Practice and the principles of the Declaration of Helsinki. Data were obtained through the electronic registries of the Galician surveillance system by personnel independent of data analysis and were anonymized before analysis. Informed consent was not required, as determined by the ethics committee.

## Results

During the study period, 13,320 (92%) of 14,476 eligible infants, received nirsevimab [5]. Seventy-seven patients were hospitalized with a positive RSV result. Eight hospitalizations were excluded (non-RSV-related (*N* = 5) and non-LRTI (*N* = 3)). Median age was 2.7 (1.5–5.2) months, 39 (56.5%) were male, and 16 (23.2%) had a high-risk condition. Demographic and clinical characteristics are represented in Table 1. Of the 69 cases (0.5% of the entire population), 45 (65.2%) were breakthrough cases who had received nirsevimab a median of 46 (minimum 9–maximum 135) days before hospitalization. Fifty percent (12/24) of the non-breakthrough patients received nirsevimab but did not meet the criteria for breakthrough case: five (20.8%) had received nirsevimab less than 7 days before symptoms onset, and seven (29.2%) had received it after hospitalization. A sensitivity analysis excluding these patients showed the results not to vary (data not shown).

**Table 1:** Characteristics of the study group and comparison between breakthrough and non-breakthrough cases. Analysis considering all included patients (%(*n*/*N*))

	Total cases (*n* = 69)	Non-breakthrough (*n* = 24)	Breakthrough (*n* = 45)	*p*-value
Sex (%(*n*/*N*))				
Male	56.5 (39/69)	62.5 (15/24)	53.3 (24/45)	0.611^a^
Female	43.5 (30/69)	37.5 (9/24)	46.7 (21(45)	-
Age at admission in months (median (IQR))	2.7 (1.5–5.2)	3.8 (1.5–5.4)	2.2 (1.5–4.8)	0.476^b^
Age at admission categorized (%(*n*/*N*))				
≤ 1 month old	11.6 (8/69)	8.3 (2/24)	13.3 (6/45)	0.487^a^
1 month to ≤ 3 months old	40.6 (28/69)	33.3 (8/24)	44.4 (20/45)	-
> 3 months old	47.8 (33/69)	58.3 (14/24)	42.2 (19/45)	-
Immunization cohort (%(*n*/*N*))				
Catch-up group	58.0 (40/69)	83.3 (20/24)	44.4 (20/45)	0.002^a^
Seasonal group	42.0 (29/69)	16.7 (4/24)	55.6 (25/45)	-
Gestational age (weeks) (median (IQR))	39 (38–40)	39.5 (38–40)	39 (37–40)	0.253^b^
Weight at birth (grams) (median (IQR))	3345 (2950–3640)	3500 (3008.8–3695)	3330 (2950–3640)	0.562^b^
Any high-risk condition (%(*n*/*N*))	23.2 (16/69)	12.5 (3/24)	28.9 (13/45)	0.147^a^
Congenital heart disease	4.4 (3/69)	0 (0/24)	6.67 (3/45)	0.547^a^
Neuromuscular diseases	1.5 (1/69)	0 (0/24)	2.2 (1/45)	1^a^
Cystic fibrosis	1.5 (1/69)	0 (0/24)	2.2 (1/45)	1^a^
Bronchopulmonary dysplasia	1.5 (1/69)	0 (0/24)	2.2 (1/45)	1^a^
Prematurity (< 37 weeks)	14.5 (10/69)	12.5 (3/24)	15.6 (7/45)	1^a^
Oxygen support (%(*n*/*N*))	63.8 (44/69)	75 (18/24)	57.8 (26/45)	0.194^a^
Intensive care unit admission (%(*n*/*N*))	21.7 (15/69)	12.5 (3/24)	26.7 (12/45)	0.229^a^
Non-invasive mechanical ventilation (%(*n*/*N*))	15.9 (11/69)	12.5 (3/24)	17.8 (8/45)	0.736^a^
Invasive mechanical ventilation (%(*n*/*N*))	0 (0/69)	0 (0/24)	0 (0/45)	-
Death (%(*n*/*N*))	0 (0/69)	0 (0/24)	0 (0/45)	-
Length of hospital stay (days), median (IQR)	4 (3–6)	4.5 (3–7)	4 (3–6)	0.252^b^
Length of ICU stay (days), median (IQR)	4 (3–5)	5 (3.5–7)	4 (3–5)	0.769^b^
RSV-associated pneumonia (%(*n*/*N*))	5.8 (4/69)	4.2 (1/24)	6.7 (3/45)	1
Co-infection/co-detection of any other pathogen in addition to RSV (%(*n*/*N*))	29 (20/69)	33.3 (8/24)	26.7 (12/45)	0.587^a^
Viral coinfection (%(*n*/*N*))	26.1 (18/69)	33.3 (8/24)	22.2 (10/45)	0.391^a^
Rhino/enterovirus* coinfection (%(*n*/*N*))	20.3 (14/69)	29.2 (7/24)	15.6 (7/45)	0.217^a^

^a^*Χ*^2^ test *p*-value. ^b^ Wilcoxon test *p*-value.*Indistinguishable in the microbiological technique used (polymerase chain reaction). *IQR*, interquartile range. Numbers are presented as percent (*n*/*N*) except where otherwise stated

The first hospitalizations occurred in September in the non-breakthrough group, and in November in the breakthrough group, with the peak in November and December, respectively. The incidence of cases was parallel to the RSV epidemic curve in both breakthrough and non-breakthrough cases. A rebound of cases at the end of the season was seen in the non-breakthrough group but not in the breakthrough cases (Supplementary Fig. 1). No VRS-LRTI hospitalizations were identified from April 16 th to May 31 st based on specific VRS-LTRI ICD- 10ES codification (J12.1, J20.5, J21.9). No differences in sociodemographic characteristics regarding nirsevimab status were observed. No significant differences were observed in the need for oxygen support, ICU admission, or NIMV. There was a significantly higher proportion of infants from the catch-up group in the non-breakthrough cases (Table 1). Another pathogen was detected in 20 (29%) of the cases. No significant differences were found in the prevalence or pattern of co-infection/co-detection between both groups (Supplementary Table 1). There were not significant differences between high-risk and non-high-risk patients with regard to clinical characteristics and course (Supplementary Table 2).

## Discussion

We describe the clinical characteristics and course of RSV-related LRTI hospitalizations in infants during the first RSV season with nirsevimab included in the immunization program in Galicia (Spain). In our setting, no significant differences were observed between breakthrough and non-breakthrough cases clinical characteristics.

Consistent with previous studies, males represented the majority of hospitalized infants, and the median age was low [12]. In contrast, the proportion of patients with high-risk conditions was notably high (20%), compared to less than 5% reported in our previous study [12]. This percentage was even higher among the breakthrough cases, where over one in four infants presented a high-risk condition.

Regarding severity, a high proportion of patients required oxygen therapy (nearly 60%), ICU admission (almost 20%), and NIMV (15%) compared with our study conducted in previous seasons [12]. In other studies conducted in other regions, where nirsevimab was implemented, also high proportions of infants requiring ICU admission (33%), oxygen support (60–88%), and NIMV (29%) were reported [6, 9, 10].

Our findings on the lack of statistically significant differences in severity markers or length of hospital stay between breakthrough and non-breakthrough cases are in line with those from other Spanish studies [6]. Contrarily, a large test-negative design study in France showed infants who had received nirsevimab needed less oxygen support than those who had not [8]. Accordingly, further larger studies are required to provide a comprehensive clinical assessment.

Our results should be interpreted with caution due to the reduced number of observations, which can limit the ability to obtain statistically significant results or even consider more complex analysis such as logistical models (for instance, the post-hoc power estimation for bivariate analysis considering having high-risk condition given the observed numbers was 39.8%). Although we only included public hospitals, historical data from the past 5 RSV seasons showed that 98% of all RSV-related LRTI hospitalizations in infants under 9 months of age occurred in public hospitals, so we can assume that we have included most hospitalizations in our cohort [2]. Breakthrough cases had received nirsevimab within the 5-month theoretical protection period. It is noteworthy that no cases occurred in this group during September and October. This could be influenced by the fact that the catch-up group was overrepresented in non-breakthrough cases and highlights the importance of an early implementation of the immunization program. It is also necessary to assess the possible waning protection of nirsevimab over the months; however, it should be taken into account that its evolution follows a pattern similar to the epidemic wave, without an increase in hospital admissions at the end of the season.

In conclusion, this study provides an initial overview of the clinical profile of RSV-related LRTI hospitalizations in the nirsevimab era. A significant proportion of the hospitalized children had high-risk conditions and disease severity markers, but the clinical course was similar in terms of the need for oxygen supplementation and/or NIMV, ICU admission, and length of stay. Further studies with larger cohorts are needed in the coming years to confirm these findings, given the anticipated decline in RSV-related LRTI hospitalizations as RSV universal prophylaxis becomes the standard of care.

## Supplementary Information

Below is the link to the electronic supplementary material.

Supplementary file1 (DOCX 15 KB)

Supplementary file2 (DOCX 2012 KB)

Supplementary file3 (DOCX 147 KB)

## Data Availability

Anonymized data will be made available upon reasonable request to the corresponding author.
