# Supplementary material for: Respiratory syncytial virus–related lower respiratory tract infection hospitalizations in infants receiving nirsevimab in Galicia (Spain): the NIRSE-GAL study
Source: Eur J Pediatr. 2025 May 2;184(5):321. doi: 10.1007/s00431-025-06151-3 (PMC12048441; doi:10.1007/s00431-025-06151-3)
Supplement: Supplementary file 1 — Supplementary file1 (DOCX 15 KB) [file 431_2025_6151_MOESM1_ESM.docx]

**Appendix 1**

Low respiratory tract infection (LRTI) was considered if the final diagnosis at discharge was any of the following (ICD-10ES):

● Viral pneumonia: J12, J12.0, J12.1, J12.2, J12.3, J12.8, J12.81, J12.82, J12.89

● Bacterial pneumonia: J13, J14, J15, J15.0, J15.1, J15.2, J15.20, J15.21, J15.211, J15.212, J15.29, J15.3, J15.4, J15.5, J15.6, J15.7, J15.8, J15.9, J16, J16.0, J16.8, J17

● Pneumonia due to unspecified microorganism: J18

● Acute bronchitis: J20, J20.1, J20.2, J20.3, J20.4, J20.5, J20.6, J20.7, J20.8, J20.9, J21, J21.0

● Asthma with acute exacerbation (if infection-induced): J45.21, J45.31, J45.41, J45.51

● Other unspecified types of asthma (Bronchial hyperreactivity/Recurrent wheezing) with acute exacerbation: J45.901

● Acute bronchiolitis: J21.1, J21.8, J21.9

● Unspecified acute lower respiratory tract infection: J22

● Unspecified bronchitis: J40
