# Supplementary material for: Respiratory syncytial virus–related lower respiratory tract infection hospitalizations in infants receiving nirsevimab in Galicia (Spain): the NIRSE-GAL study
Source: Eur J Pediatr. 2025 May 2;184(5):321. doi: 10.1007/s00431-025-06151-3 (PMC12048441; doi:10.1007/s00431-025-06151-3)
Supplement: Supplementary file 2 — Supplementary file2 (DOCX 2012 KB) [file 431_2025_6151_MOESM2_ESM.docx]

**SUPPLEMENTARY TABLES**

| Supplementary Table 1. Co-infection (or co-detection of pathogens) in RSV hospitalized patients. Distribution of patients according to the detection of additional pathogens to RSV [%(n/N)] | | | |
| --- | --- | --- | --- |
|  | **Total cases**  **(n=69)** | **Non-breakthrough**  **(n=24)** | **Breakthrough**  **(n=45)** |
| Any co-infection | 29 (20/69) | 33.3 (8/24) | 26.7 (12/45) |
| Rhino/Enterovirus* | 17.4 (12/69) | 29.2 (7/24) | 11.1 (5/45) |
| Influenza A-H1 | 1.4 (1/69) | 0 (0/24) | 2.2 (1/45) |
| Metapneumovirus | 2.9 (2/69) | 4.2 (1/24) | 2.2 (1/45) |
| Parainfluenzae + Rhino/Enterovirus | 1.4 (1/69) | 0 (0/24) | 2.2 (1/45) |
| Haemoplilus influenzae | 1.4 (1/69) | 0 (0/24) | 2.2 (1/45) |
| Bordetella Pertusis + Mycoplasma pneumoniae | 1.4 (1/69) | 0 (0/24) | 2.2 (1/45) |
| Coronavirus CoVHKU1 + Rhino/ Enterovirus | 1.4 (1/69) | 0 (0/24) | 2.2 (1/45) |
| Coronavirus NL63 | 1.4 (1/69) | 0 (0/24) | 2.2 (1/45) |

* Indistinguishable in the microbiological technique used (polymerase chain reaction). Numbers are presented as percent (n/N).

| Supplementary Table 2. Comparison between high-risk and non high-risk cases. Analysis considering all included patients | | | |
| --- | --- | --- | --- |
|  | **Non high-risk (N=53)** | **High risk**  **(N=16)** |  |
| **Sex [%(n/N)]** |  |  |  |
| Male [%(n/N)] | 60.4 (32/53) | 43.8 (7/16) | 0.264^a^ |
| Female [%(n/N)] | 39.6 (21/53) | 56.2 (9/16) |  |
| Age at admission |  |  |  |
| Median (IQR) | 1.9 (1.3-4.8) | 3.8 (2.4-6.9) | 0.074^a^ |
| Age at admission categorized [%(n/N)] |  |  |  |
| ≤1 month old | 11.3 (6/53) | 12.5 (2/16) | 0.096^a^ |
| 1< ≤3 months old | 47.2 (25/53) | 18.8 (3/16) |  |
| >3 months old | 41.5 (22/53) | 68.8 (11/16) |  |
| **Immunization cohort [%(n/N)]** |  |  |  |
| Catch-up group | 52.8 (28/53) | 75 (12/16) | 0.153^a^ |
| Seasonal group | 47.2 (25/53) | 25 (4/16) |  |
| **Gestational age (weeks). Median (IQR)** | 39 (39-40) | 35.5 (33.8-37) | <0.001^b^ |
| **Weight at birth (grams). Median (IQR)** | 3,460 (3,200-3,670) | 2,365 (2,088.5-2,996.2) | <0.001^b^ |
| **O2 support [%(n/N)]** | 67.9 (36/53) | 50 (8/16) | 0.240^a^ |
| **ICU admission [%(n/N)]** | 22.6 (12/53) | 18.8 (3/16) | 1^a^ |
| **Non-invasive mechanical ventilation [%(n/N)]** | 15.1 (8/53) | 18.8 (3/16) | 0.708^a^ |
| **Invasive mechanical ventilation[%(n/N)]** | 0 (0/53) | 0 (0/16) |  |
| **Deaths [%(n/N)]** | 0 (0/53) | 0 (0/16) |  |
| **Length of hospital stay (days). Median (IQR)** | 4 (3-6) | 3 (3-5.2) | 0.295^b^ |
| **Length of ICU stay (days). Median (IQR)** | 3.5 (2.8-5) | 5 (5-10) | 0.091^b^ |
| **Coinfection [%(n/N)]** | 28.3 (15/53) | 31.2 (5/16) | 1^a^ |
| **Viral coinfection [%(n/N)]** | 26.4 (14/53) | 25 (4/16) | 1^a^ |
| **Rhino/Enterovirus* coinfection [%(n/N)]** | 18.9 (10/53) | 25 (4/16) | 0.724^a^ |

^a^ Χ^2^ test p-value. ^b^ Wilcoxon test p-value. IQR: interquartile range. * Indistinguishable in the microbiological technique used (polymerase chain reaction). Numbers are presented as percent (n/N) except where otherwise stated.
