# Supplementary material for: Respiratory syncytial virus–related lower respiratory tract infection hospitalizations in infants receiving nirsevimab in Galicia (Spain): the NIRSE-GAL study
Source: Eur J Pediatr. 2025 May 2;184(5):321. doi: 10.1007/s00431-025-06151-3 (PMC12048441; doi:10.1007/s00431-025-06151-3)

**Supplementary figure 1. Percentage of admissions in each seasonal month, in breakthrough and no-breakthrough cases.**


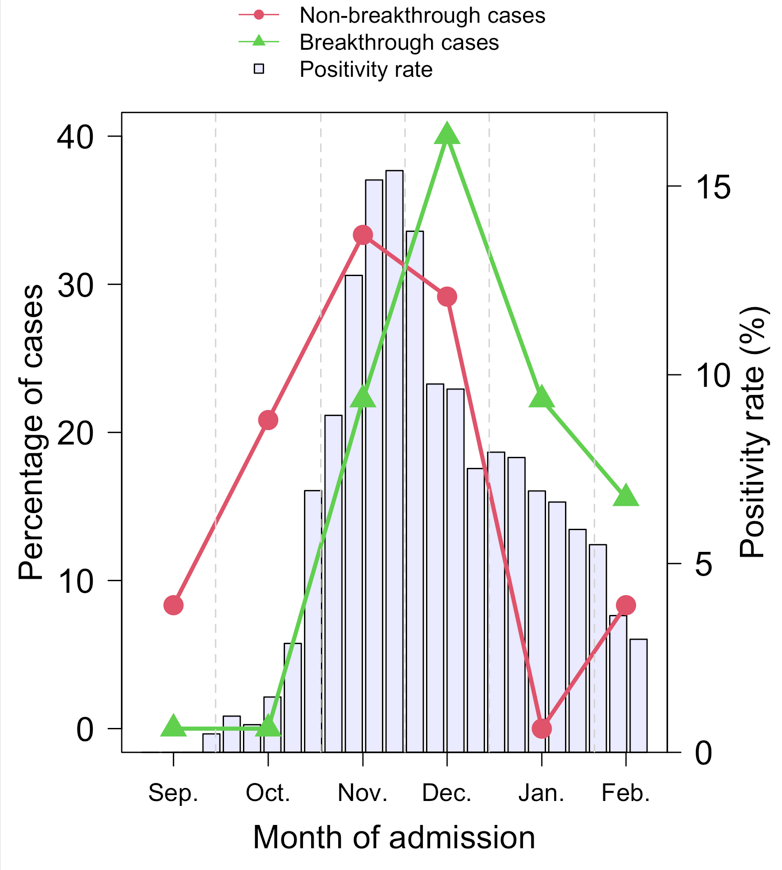

Supplement: Supplementary file 3 — Supplementary file3 (DOCX 147 KB) [file 431_2025_6151_MOESM3_ESM.docx]
